# Diversifying Robotic Ileocecectomy Methods: Superior Port Placement in a Patient With an Obstructed Crohn's Disease Flare-Up

**DOI:** 10.7759/cureus.110242

**Published:** 2026-06-04

**Authors:** Randilu Amarasinghe, Christina A Schott, Aisha Inuwa, Marian Khalili, Matthew Ng

**Affiliations:** 1 Department of Surgery, George Washington University School of Medicine and Health Sciences, Washington, DC, USA; 2 Department of Surgery, George Washington University Hospital, Washington, DC, USA

**Keywords:** crohn’s disease, ileocecectomy, minimally invasive, robotic surgery, terminal ileum

## Abstract

Crohn’s disease (CD) is an inflammatory bowel disease that can lead to fibrostenotic and fistulizing complications, requiring surgical management. We present a patient with known ileocolonic CD who presented with bowel obstruction and an interloop abscess. Imaging and intraoperative findings were consistent with advanced fibrostenotic and fistulizing disease involving the terminal ileum and adjacent small bowel.

The patient underwent a robotic-assisted ileocecectomy and small bowel resection. Due to inflammatory burden and anatomic distortion, a modified port placement strategy was used to facilitate safe dissection and resection. The surgery was successfully performed with no intraoperative or postoperative complications.

This case highlights the importance of individualized operative planning in complex CD and demonstrates the utility of adaptable robotic port placement in managing advanced intra-abdominal inflammatory disease.

## Introduction

Crohn’s disease (CD), an inflammatory bowel disease, often arises from an unknown origin. Classic cases are localized to the gastrointestinal tract; however, over 20% of patients present with extraintestinal or systemic symptoms. CD symptoms are not always benign and can lead to complications, including strictures [1,2]. Stricture formation is a sign of disease progression and is caused by the growth of the smooth muscle layer and increasing fibrosis. Thickening of the bowel wall can then progress to small bowel obstruction [3]. Fistulization of the intestinal tract is another common complication and can be further classified as simple or complex. Simple fistulas follow a single tract and occupy less than one-third of the external anal sphincter. In contrast, complex fistulas involve >30% of the external anal sphincter and span multiple tracts [4,5]. CD complications are typically treated using a multimodal approach involving both medical and surgical interventions. 

Minimally invasive surgery (MIS), such as robotic and laparoscopic approaches, has proven to be efficacious in the management of CD. A review of >5,000 patients from the National Surgical Quality Improvement Program (NSQIP) who underwent ileocecal resection demonstrated the benefits of MIS, including reduced reoperation rates, wound infection rates, and anastomotic leaks [6]. The MIS approach can sometimes be arduous, with necessary conversion to open surgery in complex cases with adhesions, abscesses, or fragile mesentery [7]. Despite these difficulties, robotic surgery for ileocolic resection has been shown to be a viable option, with shorter hospital stays and fewer 30-day postoperative complications [8]. In this report, we describe a seemingly unconventional robotic ileocecectomy in a patient with a CD flare-up.

This article was previously presented as a poster presentation at the 2024 ASCRS Annual Scientific Meeting on June 2, 2024.

## Case presentation

**Figure 1:**
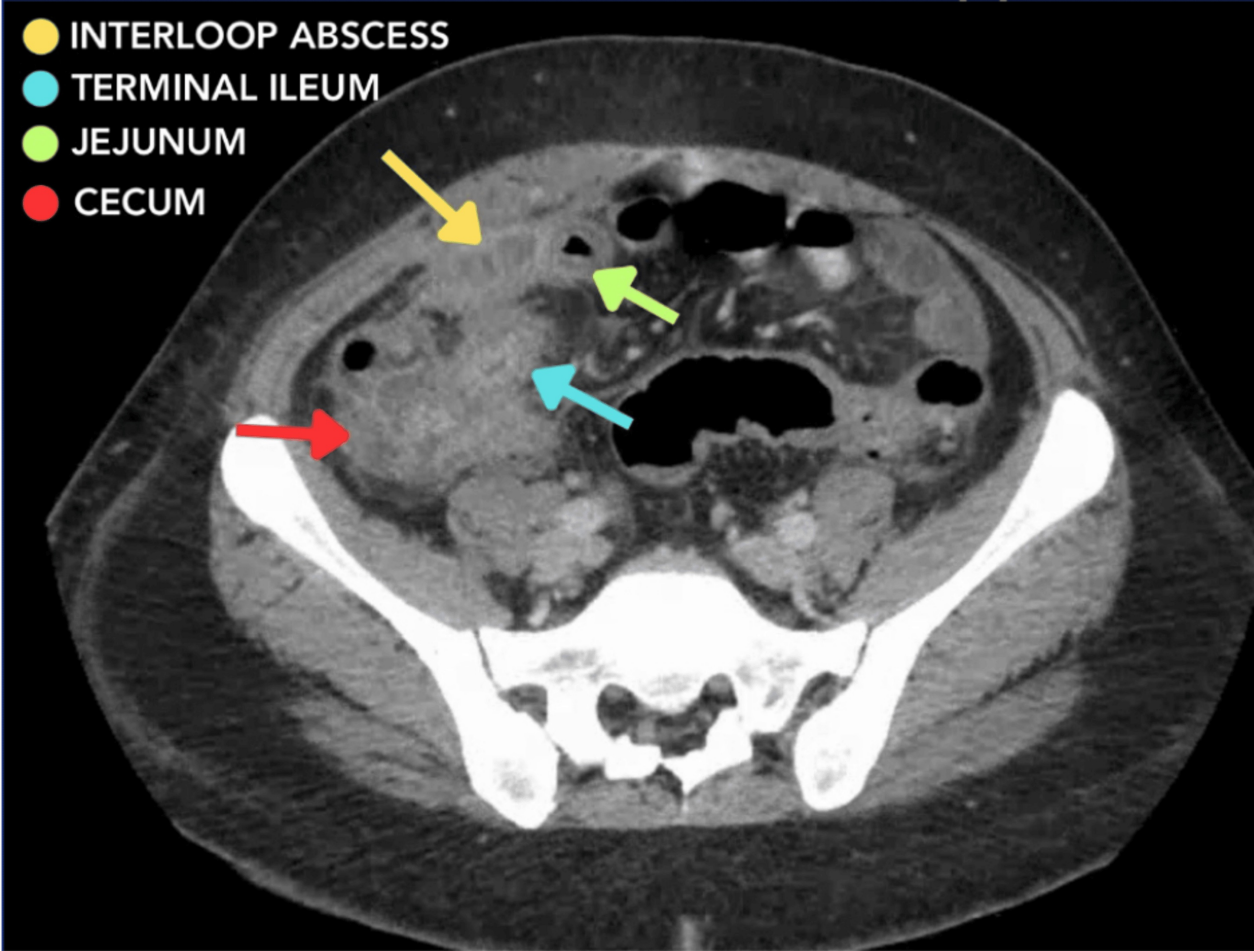
Axial view of patient’s presurgical CT of the abdomen and pelvis

A 30-year-old female patient with known CD on immunosuppressive therapy presented with 10 days of ongoing right lower quadrant abdominal pain, coupled with intermittent vomiting and decreased appetite. CT of the abdomen and pelvis demonstrated significant thickening of the gastrointestinal tract, including the cecum, terminal ileum, and appendiceal base. There was additional surrounding inflammation and a 3.7 cm abscess. The patient did not have peritonitis and was hemodynamically stable, which prompted medical management with antibiotic therapy alone. Despite the antibiotic regimen, the patient experienced worsening pain and symptoms in the following days. A repeat CT demonstrated worsening disease burden, as she developed a terminal ileum stricture, small bowel obstruction with interloop abscesses (up to 4.8 cm), and fistulous tracts (Figure 1). This disease progression required a more aggressive treatment approach through surgical intervention. 

**Figure 2:**
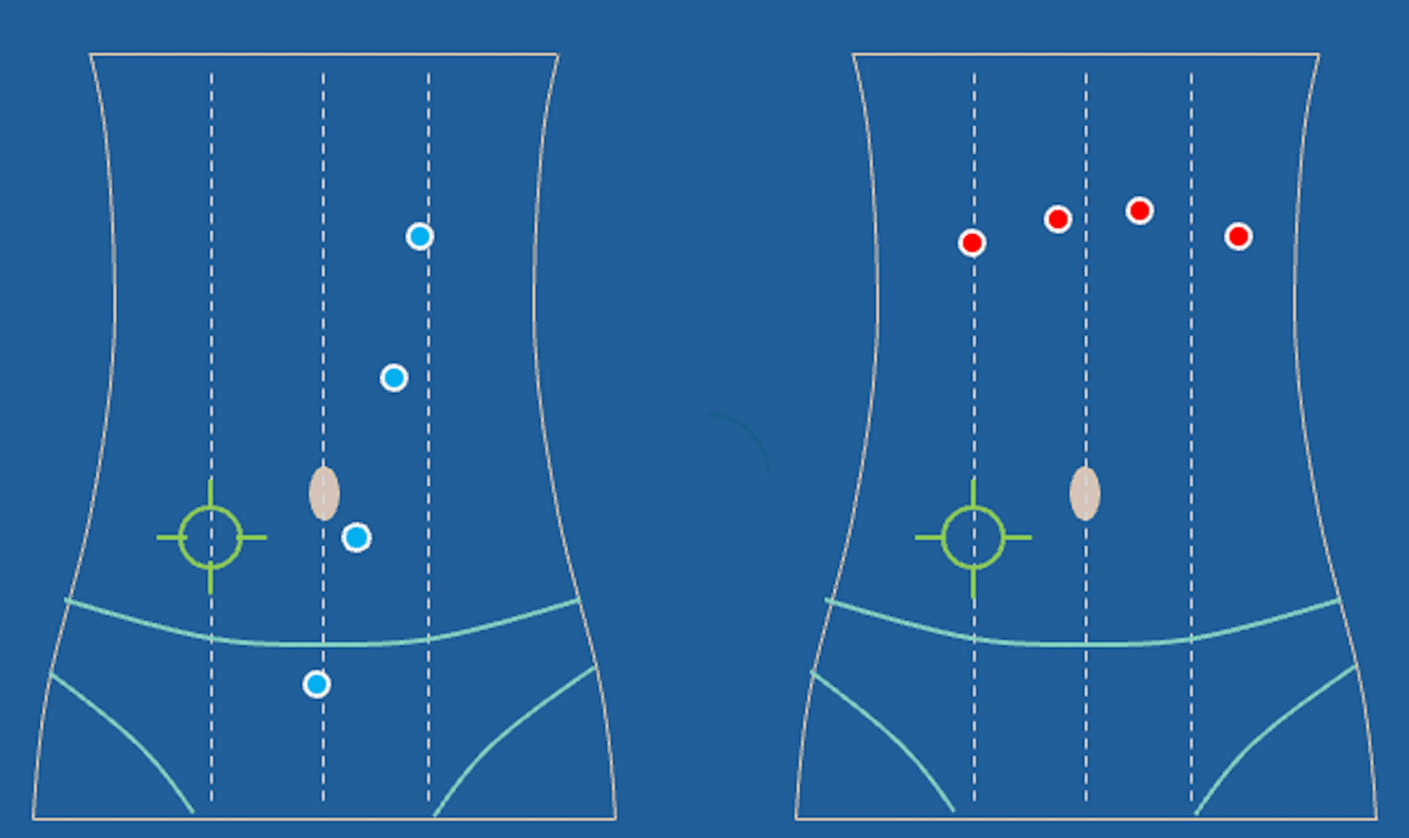
Novel port placement: left (common approach) and right (new, superior approach) This image is an original author-created schematic using PowerPoint (Microsoft® Corp., Redmond, WA, USA) and was not generated using AI.

**Figure 3:**
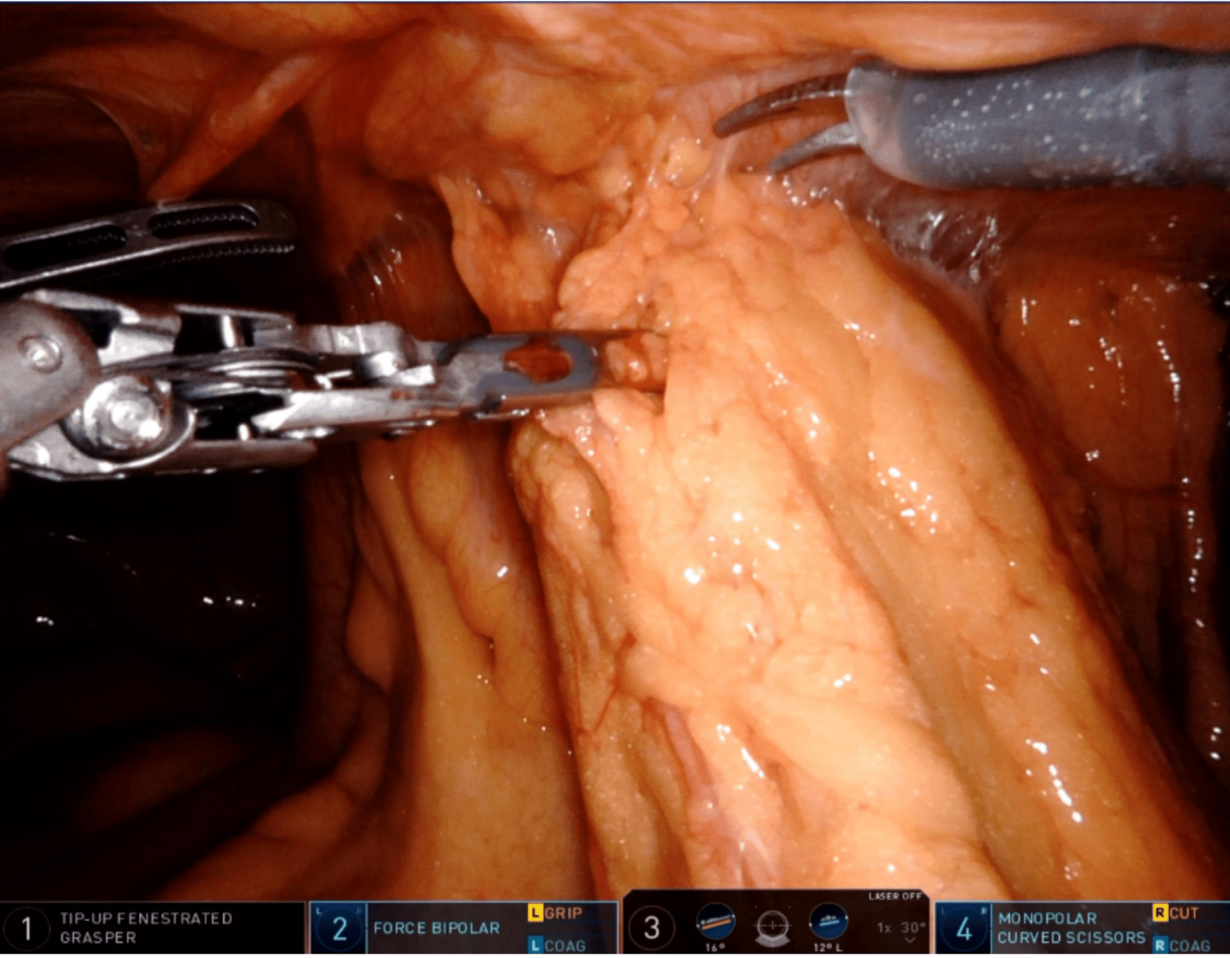
Adhesiolysis of adhered bowel to the anterior surface of the abdomen

A robotic approach was adopted; thus, a robotic ileocecectomy with ileocolonic anastomosis and a small bowel resection was performed. The patient’s disease progression posed challenges to the standard robotic procedure. The small bowel obstruction and the significant abscess extending into the abdominal wall prevented the typical placement of trocars. To provide the best access for dissection and mobilization (medial to lateral), the trocars are commonly oriented linearly, extending from the left upper quadrant to the suprapubic region. Given the multiple complications, the trocars were instead placed superiorly and oriented transversely, from the right upper quadrant to the left flank (Figure 2). This novel approach not only provided optimal exposure but also circumvented the dilated small bowel loops and large abscess, which, overall, decreased the risk of bowel injury. After adhesiolysis (Figure 3), a robotic ileocecectomy with intracorporeal ileocolonic anastomosis was performed. The dissection was performed using a lateral-to-medial and inferior-to-superior approach. There was minimal blood loss. The procedure was successful and did not require conversion to an open surgery. 

The patient recovered as expected postsurgically. As expected, a postoperative ileus was managed and she was treated with an antibiotic regimen for the abscess. After completion of the course, she was tolerating oral intake and was discharged home.

The pathology report showed inflammation of the cecum and appendix with serosal adhesions and acute ulcerations and abscess formation. The terminal ileum changes were consistent with her CD diagnosis.

Histopathologic slides were not available for inclusion in this report, despite attempts to obtain them, representing a limitation in the visual characterization of this case.

## Discussion

Knowledge of inflammatory bowel disease, and CD specifically, has been growing over recent years. Despite not knowing the exact cause of CD, it is known that it can culminate in incredibly serious complications. Identifying CD at a young age can assist in addressing patients’ symptoms; however, those diagnosed early in life present a 45% increased lifetime risk for complications such as abscesses or fistulas. A similar increase in risk applies to extraintestinal or systemic manifestations [9]. The need for surgery increases dramatically each year after diagnosis. The risk of surgery begins at about 16% in the first year after diagnosis, which then increases to about 46% after 10 years [10].

Robotic surgery can be useful, especially in patients with a narrower pelvis. It also allows for a better field of vision with limited disturbance of nearby organs. Although operation time is shown to be longer with the robotic method, the overall return to surgery (for the same indication) and hospital length of stay are shorter compared with laparoscopic and open procedures [6]. Emerging data on the practice continue to suggest that robotic surgery is a viable option on a case-by-case basis.

Our particular case demonstrates the significance of adaptability, especially in scenarios of unique presentation. As aforementioned, CD can present with a multitude of additional symptoms and complications, which can prevent surgeons from operating with their most routine techniques. One minor, yet well-known, complication with robotic port placement includes burning of the tissue surrounding the site [11]. While this superior approach was not typical, it proved to be effective in treating this patient’s CD flare-up. 

## Conclusions

CD progression can obscure common surgical steps, especially with the presence of adhesions, strictures, abscesses, fistulas, etc. In this report, we present a case of CD complicated by stricture, fistula, and abscesses that required a novel technique based on the surgeon's clinical decision-making for small bowel resection. This patient’s scenario demonstrates the importance of adaptability and tailoring a surgical approach based on the patient’s needs. 
